# Clinical Outcomes of Sonidegib in Vismodegib‐Exposed Locally Advanced Basal Cell Carcinoma: Insights From a Multicenter Descriptive Study

**DOI:** 10.1155/jskc/9016298

**Published:** 2026-02-06

**Authors:** Alvaro Prados-Carmona, María Martínez-Pérez, J. Pablo Velasco-Amador, Francisco M. Almazán-Fernández, Ricardo Ruiz-Villaverde

**Affiliations:** ^1^ Department of Dermatology, Hospital Universitario San Cecilio, Granada, Spain, juntadeandalucia.es; ^2^ Ibs, Instituto Biosanitario de Granada, Granada, Spain, ibshyderabad.org; ^3^ Hospital Pharmacy, Hospital Universitario San Cecilio, Granada, Spain, juntadeandalucia.es

**Keywords:** basal cell carcinoma, clinical outcomes, difficult-to-treat basal cell carcinoma, hedgehog pathway, locally advanced basal cell carcinoma, retrospective study, sonidegib, vismodegib exposition

## Abstract

**Background:**

Locally advanced basal cell carcinoma represents a therapeutic challenge, especially if already exposed to hedgehog (Hh) pathway inhibitor (HPI) therapy. Effectiveness after switching to another HPI still requires exploration.

**Methods:**

This observational, descriptive study retrospectively evaluates the clinical outcomes of sonidegib in patients with vismodegib‐treated locally advanced basal cell carcinoma in a real‐world, multicenter cohort across nine centers. Twelve patients with histologically aggressive locally advanced basal cell carcinoma previously exposed to vismodegib for at least 3 months were analyzed. Each received sonidegib at a standard dosage. Treatment response was assessed clinically according to tumor size changes and classified as complete response (100% decrease), partial response (at least 50% decrease), stable disease (up to 20% increase or less than 50% decrease), or disease progression (at least 20% increase). Adverse events (AEs) were documented.

**Results:**

Two patients achieved complete clinical response, nine demonstrated partial response, and one exhibited disease progression. Disease control was achieved in 11 of the 12 patients (91.7%). No novel AEs have been documented, and they were managed through dose adjustments or temporary interruptions.

**Conclusion:**

These findings suggest that sonidegib could still show efficacy and may still serve as a viable second‐line option after prior Hh pathway suspension.

**Plain Language Summary**
• This study retrospectively evaluates the clinical outcomes of sonidegib in patients with locally advanced basal cell carcinoma that had already been exposed to vismodegib, in a real‐world, multicenter cohort.
• In the analyzed cohort, sonidegib proved beneficial for a majority of patients, enabling disease control without novel adverse events. If confirmed by ad‐hoc designed, prospective studies, sonidegib could represent a promising therapeutic option for patients with challenging disease (refractory or intolerant to previous vismodegib).
• These findings suggest that some hedgehog pathway inhibitors could still show efficacy and may serve as a viable second‐line option after prior hedgehog pathway inhibitor suspension. Continued research into the mutations that lead to treatment resistance will be crucial in refining treatment strategies and improving outcomes by individualizing treatment approaches.

## 1. Introduction

Basal cell carcinoma (BCC) is the most prevalent type of skin cancer worldwide, primarily arising in photoexposed areas. It is frequently managed with localized therapies. However, a subset of patients develops locally advanced or difficult‐to‐treat BCC (laBCC), characterized by significant local invasion that precludes effective surgical or radiotherapeutic management [1, 2]. In these cases, systemic treatments targeting tumorigenic pathways, notably the hedgehog (Hh) signaling pathway, have emerged as critical therapeutic options [3].

The Hh pathway plays a pivotal role in BCC pathogenesis, and its dysregulation, often through mutations in the *PTCH1* or smoothened (SMO) genes, contributes to uncontrolled cellular proliferation. Vismodegib, a first‐in‐class Hh pathway inhibitor (HPI), was approved as systemic treatment option for laBCC and metastatic BCC (mBCC), demonstrating significant tumor control in clinical trials and real‐world settings [4, 5]. Despite its efficacy, drug intolerance or uncomplete response to vismodegib, whether through primary nonresponse or acquired mechanisms, limits its long‐term success in some patients [5].

Sonidegib represents an alternative HPI, acting similarly through SMO inhibition but offering unique pharmacokinetic properties and potential efficacy advantages [6–8]. Sonidegib was evaluated in the pivotal BOLT trial, where it demonstrated objective response rates (ORRs) comparable to those of vismodegib, with a tolerable safety profile conducive to long‐term management [9, 10]. However, evidence regarding sonidegib’s effectiveness specifically in patients with prior vismodegib exposure remains limited [8]. This gap in knowledge is especially relevant in real‐world settings, as the differences in their tolerability profile could eventually prompt a switching from one therapy to another.

This retrospective study aims to further assess the clinical outcomes of sonidegib in a multicenter cohort of patients with laBCC who received prior treatment with vismodegib [11]. By examining response rates, disease control, and adverse events (AEs) in this patient subset, the study seeks shed more light on second‐line therapy options for laBCC, aligning with an evolving therapeutic landscape focused on individualized and, if needed, sequential treatment strategies.

## 2. Methods

This study analyses the published dataset of an observational, descriptive, retrospective longitudinal, multicenter study, conducted across nine Dermatology centers in Andalusia, Spain, encompassing patients treated between 1^st^ January 2021 and 1^st^ January 2023. The study protocol adhered to the principles outlined in the Declaration of Helsinki and relevant national regulations and received approval by the pertinent Institutional Review Board (Ethics Committee of Hospital Universitario San Cecilio, Granada, Spain, approval code: HUSC_DERM_008/2023). Informed consent was obtained from all participants for the use of their anonymized clinical data in research [11].

### 2.1. Patient Selection

Inclusion criteria consisted of adult patients (≥ 18 years) with histologically confirmed laBCC who had to suspend previous vismodegib therapy. Suspension was decided according to clinical effectivity criteria, considering progressive disease (PD) or stable disease (SD) according to the criteria used for response assessment described in the next section.Namely, PD when the tumor increased over 20% of its original size and SD when there was a size increase smaller than 20% of original size or, in the best of cases, limited clinical improvement with decreases of less than 50% of original size after a minimum of 3 months of vismodegib treatment. Patients with basal cell nevus syndrome could enroll if they met inclusion criteria. Exclusion criteria involved patients with prior sonidegib exposure or those receiving concurrent systemic therapies for other malignancies.

### 2.2. Treatment Protocol

All patients received sonidegib (Odomzo, Sun Pharmaceutical Industries Europe B.V, Hoofddorp, Netherlands) for at least 3 months for locally advanced BCC (European Association of Dermato‐Oncology [EADO] classification stage III) at a standard dose of 200 mg once daily according to technical data sheet. Treatment continued until disease progression, stabilization of response, intolerable AEs, or voluntary withdrawal. Dose adjustments, including temporary interruptions or reductions to every‐other‐day dosing, were allowed for managing AEs, following institutional guidelines and the product’s prescribing information.

### 2.3. Outcomes and Response Assessment

The primary outcome was the ORR, defined as the sum of complete responses (CRs) and partial responses (PRs) based on clinical and, if needed, imaging criteria. Tumor response was assessed, at least, every 3 months through physical examination and, where applicable, imaging studies (e.g., MRI or CT), according to the parameters of the previously published Spanish series [11, 12], considering CR when there was no visible tumor; PR when the tumor decreased by at least 50% in size; SD when tumor reduction was less than 50%, or less than 20% increase; or PD, when the tumor increased in size by at least 20%.

### 2.4. Safety Monitoring and AE Management

AEs were systematically recorded at each follow‐up visit and graded according to the Common Terminology Criteria for Adverse Events (CTCAE) v5.0. The management of AEs involved dose modifications or treatment interruptions when necessary.

### 2.5. Data Analysis and Manuscript Writing

Descriptive statistics were used to summarize patient demographics, response rates, and AEs. Measures of central tendency have been reported alongside their corresponding measures of dispersion (e.g. mean [± standard deviation]). Where relevant, minimum and maximum values have also been provided. Confidence intervals (CIs) have been calculated at the 95% level, and all statistical tests were two‐sided, with significance set at *p* < 0.05. All analyses were conducted using SPSS v.25 (SPSS, Chicago, IL, USA). During the preparation of this work, the authors used ChatGPT‐4o (OpenAI, California, USA) with human oversight to improve readability of the work. After using this tool, the authors reviewed and edited the content as needed and take full responsibility for the content of the publication.

## 3. Results

**Figure 1:**
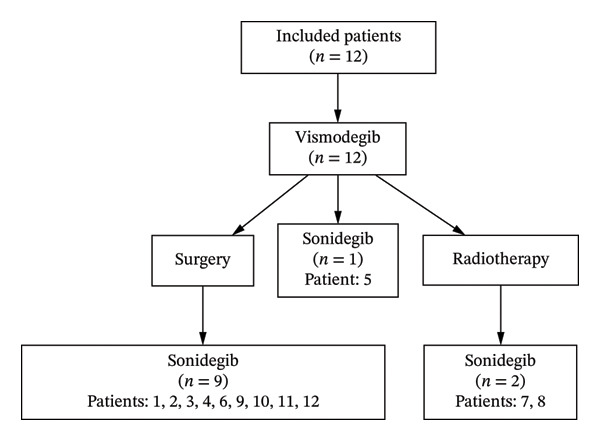
Flowchart of previous therapies of patients included in the study.

A total of 12 patients with laBCC previously exposed to vismodegib therapy were included in the study (Figure 1). The median duration of prior vismodegib treatment was 6.7 months (range: 3–14 months). Vismodegib was discontinued due to PD or SD at the discretion of the prescribing physician or multidisciplinary committee, with concomitant side effects not tolerated by the patient in 25% of the cases. All the patients showed aggressive histologic subtypes (infiltrating pattern of growth, morpheiform, micronodular, or basosquamous). No patients needed to be excluded from the analysis for presenting exclusion criteria [11].

The median age was 75.5 years (range: 40–93 years), with a sex distribution of 50% male and 50% female (Table 1). Comorbidities were present in 41.66% of the patients, with the most common being hypertension (*n* = 2) and obesity (*n* = 2).

**TABLE 1:** Baseline characteristics and treatment outcomes.

ID	Age (years)	Sex	Comorbidities	Histologic subtype	Previous treatment (if any)	Months to initial response	Months to definitive response	Clinical outcome	Disease controlled
1	58	Woman	Gorlin syndrome, acromegaly, neurofibromatosis, polycystic kidney disease, liver transplant	Aggressive	Vismodegib surgery	Unknown	Unknown	CR	Yes
2	74	Woman	Hypertension, neurinoma, obesity	Aggressive	Vismodegib surgery	11	11	PR	Yes
3	79	Man	Chronic kidney disease, hypertension, obesity	Aggressive	Vismodegib surgery	2	9	PR	Yes
4	73	Man	Non‐Hodgkin lymphoma (in remission)	Aggressive	Vismodegib surgery	1	6	CR	Yes
5	40	Man	Unknown	Aggressive	Vismodegib	1	3	PR	Yes
6	79	Man	Unknown	Aggressive	Vismodegib surgery	2	2	PR	Yes
7	77	Woman	Melanoma	Aggressive	Vismodegib radiotherapy	3	13	PR	Yes
8	73	Man	Unknown	Aggressive	Vismodegib radiotherapy	3	6	PR	Yes
9	83	Man	Unknown	Aggressive	Vismodegib surgery	3	9	PR	Yes
10	84	Woman	Unknown	Aggressive	Vismodegib surgery	4	13	PR	Yes
11	66	Woman	None	Aggressive	Vismodegib surgery	6	18	PR^∗^	Yes
12	93	Woman	None	Aggressive	Vismodegib surgery	No response	No response	DP	No

*Note:* Aggressive histologic subtypes included those BCC with infiltrating pattern of growth, morpheiform, micronodular, or basosquamous histotypes. Response was assessed clinically as follows: complete response (CR) when there was no visible tumor; partial response (PR) when the tumor decreased by at least 50% in size; stable disease (SD) when tumor reduction was less than 50%, or less than 20% increase in tumor area; and disease progression (PD), when the tumor increased in size by at least 20%.

^∗^Patient 11 received pembrolizumab 2 mg per kilogram of weigh every 3 weeks and radiotherapy while being treated with sonidegib for her laBCC as part of an ongoing research project.

### 3.1. Treatment and Response Rates

**Figure 2:**
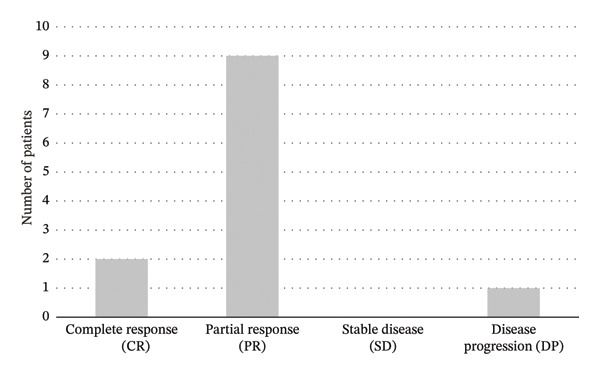
Distribution of treatment responses to sonidegib in vismodegib‐exposed laBCC. Complete response (CR) was considered when there was no visible tumor (reduction of 100% of its original size); partial response (PR) when the tumor decreased by at least 50% in size; stable disease (SD) when tumor reduction was less than 50%, or less than 20% increase; or progressive disease (PD), when the tumor increased in size by at least 20%.

Following initiation of sonidegib therapy at 200 mg daily, 2 patients (16.7%) achieved a CR, and 9 patients (75%) demonstrated PR, resulting in an ORR of 91.7%. One of the patients that showed PR received concomitant treatment with pembrolizumab 2 mg per kilogram of weigh every 3 weeks and radiotherapy for his laBCC as part of an ongoing research project at the institution involved in her care. Only one patient (8.3%) experienced PD despite sonidegib treatment. Distribution of treatment responses is represented in Figure 2. Mean time to objective response among responders was 8.18 months (± SD 5.24). Mean time to initial response was 3 months (± SD 2.97).

### 3.2. Safety and AEs

**Figure 3:**
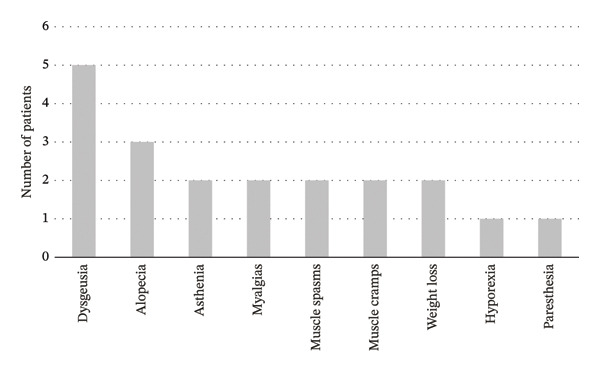
Distribution of adverse effects in the population treated.

AEs were reported in 100% of the patients, with the most common AEs being dysgeusia (*n* = 5) and alopecia (*n* = 3). Others include asthenia (*n* = 2), myalgias (*n* = 2), muscle spasms (*n* = 2), weight loss (*n* = 2), muscle cramps (*n* = 2), hyporexia (*n* = 1), and paresthesia (*n* = 1). AEs are shown in Figure 3. Dose adjustments were implemented in 75% of the patients due to AEs, with temporary interruptions (*n* = 5) or reductions (*n* = 5) to every‐other‐day dosing where necessary. No grade 4 or 5 AEs were observed. All the patients successfully continued treatment with manageable AEs.

### 3.3. Summary of Key Findings

Sonidegib demonstrated a high ORR (91.7%) in the examined dataset of patients. Despite the high incidence of AEs, the tolerability profile with dose reductions as per technical data sheet supported sonidegib as a viable therapeutic option, even for patients who had to suspend or failed to obtain complete response after initial HPI therapy with vismodegib.

## 4. Discussion

This study assessed the efficacy and safety of sonidegib as a second‐line therapy in a subset of patients from a published dataset considered to have unsuccessfully treated laBCC after prior vismodegib treatment (SD or PD) by the authors. With an ORR of 91.7%, including CR in 16.7% of patients (*n* = 2) and PR in 75% (*n* = 9), sonidegib showed promising efficacy. The disease control rate (DCR) and the median time of treatment described is similar to the data shared in other real‐world retrospective series [10, 13]; however, the key difference is that these pool of patients had been exposed to vismodegib treatment with a response deemed suboptimal by the prescribing physician or multidisciplinary committee. These findings would support its role as an effective therapeutic alternative for this challenging subset of patients, providing additional options for disease control in cases of vismodegib insufficient response or intolerance [6].

### 4.1. Mechanisms of Resistance and Sequential HPI Therapy

Inactivating mutations of PTCH1 on chromosome 9q22.3 are identified in a majority of sporadic BCCs, while activating mutations of the pathway activator SMO and suppressor of fused (SUFU) are also observed [9, 14]. Sonidegib (LDE225) is an oral HPI that selectively targets SMO, thereby inhibiting Hh pathway signaling [15]. It is the second drug, after vismodegib, approved for laBCC through this mechanism [8]. It has demonstrated its efficacy and safety in patients with laBCC in the Phase II BOLT trial, where 61% (95% CI: 48; 72) of patients treated with 200 mg of sonidegib achieved an objective response to treatment, with a median time to response of four months [16, 17].

Both primary and secondary HHI resistance mechanisms involve mutations in SMO impairing drug binding or activating SMO at different levels. Secondary resistance also involves copy number changes in SUFU and Gli2 [14]. Approximately, 20% of initial responders to vismodegib have been reported to potentially develop resistance [18–20]. One of the postulated mechanisms underlying resistance to vismodegib is the presence of mutations in the SMO gene [21, 22]. Acquired SMO mutations maintain Hh signaling by either impairing drug binding or inducing constitutive activity of SMO [18]. Sonidegib also blocks Hh signaling by selective inhibition of SMO, but its chemical structure is different from vismodegib [23]. Some SMO mutations have also been shown to display functional resistance to sonidegib in vitro [18, 22]. An investigator initiated open‐label study was conducted to assess the tumor response to sonidegib in patients with an advanced BCC that previously was resistant to treatment with vismodegib, suggesting similar treatment resistance to sonidegib [18]. However, the evidence is still limited. Certain mutations might confer resistance specifically to vismodegib, potentially leaving a therapeutic window for alternative agents like sonidegib. One of the limitations of the aforementioned study [18] is that the authors only analyzed 9 patients who were being treated with 800 mg sonidegib daily for a median duration of treatment of just 6 weeks. Despite this dose regime has been tested in the BOLT clinical trial, the safety and tolerability profiles of sonidegib 200 mg proved to be more favorable than those for sonidegib 800 mg, with lower overall incidences in each AE category [9, 17]. AEs in the 200 mg group were primarily Grade 1 or 2, mostly manageable and reversible with dose interruptions without overall impact on efficacy. On the other hand, the 800 mg daily dose led to more treatment withdrawals without a higher ORR, noticing that patients in the 200 mg group were more likely to remain on treatment for longer periods. This is the key as the median time to tumor response for the 800 mg group was 3.8 months [9]. Taking this into consideration, it is important to note the early discontinuation of sonidegib in the referred investigator‐initiated study (4 out of 9 patients were treated with sonidegib for 5 weeks or less). Additionally, one of the nine subjects was not evaluable. As the authors themselves suggest, this could have led to inability to detect a response. Nevertheless, 3 patients experienced stable disease with a median of only 4 weeks of treatment with sonidegib (range: 3–7 weeks) and two subjects elected to discontinue sonidegib for surgery (which, we do not know, may or may not had been possible before sonidegib). Interestingly, this happened although functional resistance in vitro to either sonidegib or vismodegib were identified in five of eight available baseline tumor samples and, what is more, 1 patient with an SMO mutation demonstrated maintaining stable disease for 58 weeks before eventual disease progression [18].

Our results could align with the hypothesis that not all SMO mutations equally impact the efficacy of sonidegib, allowing it to retain activity where vismodegib may fail. This observation correlates with other previously reported cases [24] and correlates with the NCCN guidelines statement referring to the ongoing research on newer HPI to see if they can provide higher rates of response, more durable responses, responses in less advanced BCC, or responses in BCC resistant to vismodegib [3]. As potential genetic vismodegib resistance mechanisms were not formally confirmed, adaptive resistance pathways cannot be excluded. Additionally, ours is still a small sample, but the differences found compared with the previous investigator‐initiated trial might rely on the longer treatment period [18]. Some factor to consider regarding the success of sonidegib in our sample could be in each drug’s pharmacokinetics and pharmacodynamics. Sonidegib unique pharmacologic profile has already been explored in depth, demonstrating interindividual pharmacokinetic variability and even a clinically significant impact of dietary intake on drug exposure [25]. Despite that, its higher volume of distribution (9170 L for sonidegib vs. 16.4–26.6 L for vismodegib) might have led to higher cutaneous distribution, and the prolonged half‐life of the drug (28 days for sonidegib vs. 4 days for vismodegib) enabled dose adjustments and temporary interruptions for a better tolerance [7, 8, 26–28]. As a consequence, better tolerability and longer treatment periods may be the main reason for these results considering that no mutational analysis has been carried out in our patients.

### 4.2. Comparative Efficacy With Prior Studies

Real‐world evidence has shown that sonidegib has both efficacy and adequate tolerability, making it viable for long‐term management. The response rates observed in our study are consistent with data from other real‐world series [10–12, 29]. The DCR recorded in the BOLT trial was also 91% for laBCC in the 200 mg daily dosage arm [9]. However, our study focused specifically on patients with previous vismodegib exposure. This distinction should be noted for future comparisons evaluating the utility of sonidegib as a sequential HPI therapy. Additionally, these findings underscore the need for personalized treatment strategies and further support the clinical utility of sonidegib in patients with limited alternatives.

Tolerability is a critical consideration in the treatment of elderly patients with multiple comorbidities, as is common in laBCC populations. Commonly observed AEs with these drugs were also seen in our cohort (dysgeusia, alopecia, asthenia, and muscle spasms) which aligns with sonidegib’s established safety profile in the BOLT trial and other studies [8, 11, 14]. Importantly, these AEs were manageable through dose adjustments, with no severe AEs (Grade 4 or 5) reported, allowing patients to continue treatment. This flexibility in dosing may be particularly beneficial for patients who experience intolerable side effects, providing that it does not compromise effectiveness [7, 9, 30].

### 4.3. Implications for Clinical Practice

Our findings, although they still need to be confirmed through further studies, would endorse sonidegib as a viable second‐line treatment for laBCC patients having to suspend vismodegib. The high ORR observed in this study supports the practice of sequential HPI therapy if needed in extreme cases, potentially offering meaningful clinical responses. Clinicians may consider sonidegib for patients who either fail to respond enough to or cannot tolerate vismodegib. This tailored approach is consistent with current guidelines, promoting an individualized, multidisciplinary, therapeutic planning for managing advanced BCC, situation for which no preferred regimen has been standardized [3].

### 4.4. Limitations and Future Directions

Despite these promising findings, the nature of this study requires caution for accurate interpretation. The analysis has been performed on the dataset of a retrospective study with a relatively small sample size that also limits the generalizability of the results. A total of 25% of patients included had suspended vismodegib due to AEs on top of the lack of efficacy after a minimum of 3 months (median 6.7 months [range: 3–14 months]), but lack of efficacy was deemed both in cases of disease progression (increase in 20% of original size) or stabilized disease which could include limited clinical response (decrease in less than 50% of original size by the time of withdrawal). This is relevant as in the cohort the range of months for prior exposure to vismodegib included patients with less than 6 months of exposure. Despite mean time to response for vismodegib is considered approximately 3 months both in trials and real‐life experience, improved response can be reached in the subsequent months of treatment [4, 31, 32]. Equally, response to sonidegib was not assessed using the Conventional Response Evaluation Criteria in Solid Tumors (RECIST) or the modified RECIST (mRECIST), but clinically without histological confirmation. Efficacy was determined by each prescriptor without being centrally reviewed. Wash‐out periods between treatments were not standardized. Therefore, the potential misinterpretation of response to sonidegib in cases without enough vismodegib exposition or enough wash‐out periods after vismodegib should be considered. Additionally, the lack of DNA profiling data prevents a definitive assessment of specific resistance mutations, if any, and their impact on sonidegib response. One patient who showed partial response received concomitant treatment with other therapies in addition to sonidegib that might also impact disease prognosis. Future prospective studies with larger cohorts and mutational analysis would be valuable for identifying predictive factors of response to sequential HPI therapy. Exploring molecular resistance mechanisms could provide a basis for integrating next‐generation HPIs or combination therapies, further personalizing the management of laBCC.

## 5. Conclusion

In the analyzed cohort of vismodegib‐exposed patients, sonidegib proved beneficial for a majority of patients. If confirmed by *ad-hoc* designed, prospective studies, sonidegib could still represent a promising therapeutic option for some patients with laBCC that have to suspend vismodegib, not only due to its efficacy but also because of a manageable safety profile. These findings could also support the potential for sequential HPI therapies in case of persistent unviability of surgery or radiotherapy and highlight the importance of individualized treatment approaches. Continued research into the molecular basis of HPI resistance will be crucial in refining treatment strategies and improving outcomes for this challenging patient population.

## Author Contributions

Conceptualization: Alvaro Prados‐Carmona, María Martínez‐Pérez, J. Pablo Velasco‐Amador, Francisco M. Almazán‐Fernández, and Ricardo Ruiz‐Villaverde. Data curation: Alvaro Prados‐Carmona, María Martínez‐Pérez, J. Pablo Velasco‐Amador, Francisco M. Almazán‐Fernández, and Ricardo Ruiz‐Villaverde. Writing–original draft preparation: Alvaro Prados‐Carmona, María Martínez‐Pérez, J. Pablo Velasco‐Amador, Francisco M. Almazán‐Fernández, and Ricardo Ruiz‐Villaverde. Writing–review and editing: Alvaro Prados‐Carmona, María Martínez‐Pérez, J. Pablo Velasco‐Amador, Francisco M. Almazán‐Fernández, and Ricardo Ruiz‐Villaverde.

## Funding

This article has no funding source.

## Ethics Statement

This study was approved by the Ethics Committee of Hospital Universitario San Cecilio, Granada, Spain, approval code: HUSC_DERM_008/2023.

## Consent

Written informed consent was obtained from the patients for the processing of their case details.

## Conflicts of Interest

The authors declare no conflicts of interest.

## Data Availability

The data that support the findings of this study are available from the corresponding author upon reasonable request. All the authors had full access to all the data in this manuscript and jointly assume responsibility for its integrity.
